# VARIATIONS IN SUSTAINING IMPLEMENTATION OF STAR-VA: THE ROLE OF KNOWLEDGE RESERVOIRS AND OTHER FACTORS

**DOI:** 10.1093/geroni/igz038.2355

**Published:** 2019-11-08

**Authors:** Jennifer L Sullivan, Kim Curyto, Omonyêlé l adjognon, Jacqueline Pendergast, Laura O Wray, Michele Karel

**Affiliations:** 1 Center for Healthcare Organization and Implementation Research (CHOIR), VA Boston Healthcare System, Boston, Massachusetts, United States; 2 vA western new york healthcare system, batavia, New York, United States; 3 center for healthcare organization and implementation research, boston VA healthcare system, boston, Massachusetts, United States; 4 VA Center for Integrated Healthcare, VA Western NY Healthcare System (116N), Buffalo, New York, United States; 5 Office of Mental Health and Suicide Prevention, Veterans Health Administration, Underhill, Vermont, United States

## Abstract

Variation in STAR-VA sustainability across 20 trained VA Community Living Centers was explored using prospective qualitative methods utilizing the knowledge reservoirs framework including seven domains: People, Routines, Artifacts, Relationships, Information space, Culture, and Structure. We conducted directed content analysis of transcripts to identify facilitators and barriers of successful program sustainment. We found that people, usual routines, information sharing, and team relationships were the most often mentioned facilitators by CLC staff. Common reported barriers were people, team relationships, and work culture. Overlap was found in knowledge reservoirs acting as both facilitators and barriers at the same site, most often for people/teams, team relationships, and work culture. Results will be used to develop a sustainability intervention focused on addressing reported barriers. Most notably, a focus on having the appropriate team members, positive team relationships, usual routines, and a supportive work culture are critical for STAR-VA sustainability efforts.

